# Reproductive health crisis during waves one and two of the COVID-19 pandemic in India: Incidence and deaths from severe maternal complications in more than 202,000 hospital births

**DOI:** 10.1016/j.eclinm.2021.101063

**Published:** 2021-07-29

**Authors:** Manisha Nair, MaatHRI writing group, on behalf of the MaatHRI collaborators

**Affiliations:** National Perinatal Epidemiology Unit, Nuffield Department of Population Health, University of Oxford, Oxford, UK

**Keywords:** Maternal complications, Maternal death, Septic abortion, SARS-CoV-2, India, Incidence, Case-fatality, CIs, Confidence Intervals, DIC, Disseminated intravascular coagulation, FIGO, International Federation of Gynaecology and Obstetrics, GRSI, Government Response Stringency Index, MaatHRI, Maternal and perinatal Health Research collaboration, India, PPH, Postpartum haemorrhage, RR, Rate ratios, SARS-CoV-2, Severe acute respiratory syndrome caused by the Coronavirus 2, WHO, World Health Organisation

## Abstract

**Background:**

The SARS-CoV-2 pandemic in India has adversely affected many aspects of population health. We need detailed evidence of the impact on reproductive health in India so that lessons can be learnt.

**Methods:**

Hospital-based repeated monthly survey of nine severe maternal complications and death in 15 hospitals across five states in India covering a total of 202,986 hospital births, December-2018 through to May-2021. We calculated incidence rates (with 95% confidence intervals (CIs)) per 1000 hospital births, case-fatality and rate ratios (RR) with 95% CIs. Linear regression was used to examine the association between the Government Response Stringency Index (GRSI) for India and changes in hospital births, incidence and case-fatality.

**Findings:**

There was a significant decrease in hospital births per month during the pandemic period with a 4.8% decrease per 10% increase in the GRSI scores (*p* < 0.001). The overall incidence of severe complications in the pandemic period was not significantly different from the pre-pandemic period, but hospital admissions from septic abortion was 56% higher (RR=1.56; 95% CI=1.22–1.99; *p* < 0.001). The overall case-fatality of complications increased by 23% (RR=1.23; 95% CI=1.03–1.46; *p* = 0.022) and remained high across the different phases of the pandemic with a notable significant increase in deaths from heart failure in pregnancy.

**Interpretation:**

Our study supports the legitimacy of the calls made to maintain sexual and reproductive health services as essential services during the pandemic. Lessons learnt should be used to avert the ongoing reproductive health crisis while India plans to manage a third wave of the pandemic.

**Funding:**

The MaatHRI platform and this study are funded by a Medical Research Council Career Development Award to MN (Ref:MR/P022030/1). The funder has no role in the study design, data collection, analysis, or writing the paper

**Research in context**

**Evidence before this study**

Serious concerns have been raised about the impact of lockdowns and restrictions on access to sexual and reproductive health services with predicted rise in unsafe abortions and decrease in access and demand for care. A recent systematic review showed that maternal deaths increased by 37% during the SARS-CoV-2 pandemic compared with the pre-pandemic period. Existing studies from India are unable to provide detailed evidence of the impact on reproductive health in the country and the trends across the first and second waves.

**Added value of this study**

This is the first large study from a hospital-based repeated monthly survey across five States in India comparing the incidence and case-fatality of severe maternal complications in 202,986 births during the pandemic and pre-pandemic periods over a duration of 30 months. The study not only demonstrates a decrease in hospital births and a 23% increase in case-fatality of severe maternal complications, it also showed a 56% increase in hospital admissions from septic abortion during the pandemic period. Notably, septic abortion rate increased by two-fold and 1.8-fold corresponding with the periods of rising first and second waves, respectively.

**Implications of all the available evidence**

This study supports the legitimacy of the calls made by the WHO, other international organisations and scientists to maintain sexual and reproductive health services as essential services to continue to provide high quality care to avert rise in maternal mortality and morbidity during the pandemic. While India is preparing to manage a third wave of the pandemic, the country needs to take urgent action to mitigate the ongoing reproductive health crisis using the lessons learnt from the first and second waves.

Alt-text: Unlabelled box

## 1 Introduction

The severe acute respiratory syndrome caused by the Coronavirus 2 (SARS-CoV-2) pandemic has claimed many lives across the world with many countries going through a second or third wave of infection. India recently went through a devastating second wave which started in March 2021. Until the end of May 2021, more than 27 million cases and 315,000 deaths from SARS-CoV-2 infection have been reported in India [1] and many fear that the actual numbers could be higher. In addition to its direct impact on mortality and morbidity, the pandemic has had significant indirect adverse effects on many other aspects of population health including reproductive health. A modelling study at the start of the pandemic predicted an excess of 113,400 maternal deaths per annum globally considering a scenario of severe impact on healthcare systems [2]. A recent systematic review found that maternal deaths increased by 37% during the SARS-CoV-2 pandemic compared with the pre-pandemic period [3]. However, only three studies provided information about maternal deaths, two from India [4,5] and one from Mexico [6] and the significant increase estimated in the pooled analysis was driven by the Mexico study alone. The two studies from India included a total of 4160 pregnancies during the pandemic and 11,512 pregnancies in the pre-pandemic period [3]. One study found a 7% increase in maternal deaths [5], but the other did not find an increase during the pandemic compared with the pre-pandemic period [4]. The studies only included data from the beginning of the first wave of the pandemic up to August 2020^4,5^ and were unable to provide detailed evidence of the impact of the pandemic on maternal mortality and morbidity in India as the pandemic unfolded and the trends across the first and second waves.

Serious concerns have been raised about the impact of lockdowns and restrictions on access to sexual and reproductive health services with a predicted rise in unsafe abortions [7], [8], [9] and decrease in access and demand for care [2]. These concerns are especially relevant for India, which has the second highest number of maternal deaths in the world. However, the country has been making significant progress to reduce this high burden in recent years [10]. There is currently a significant gap in understanding the impact of the pandemic on the incidence and case-fatality of severe maternal complications and in the trends of institutional births, which could be an indicator of access to healthcare services. Addressing this knowledge gap will help India, and other countries, in assessing the potential impact on the progress made in reducing maternal deaths and accordingly develop effective plans and health system interventions to mitigate adverse effects. The recent systematic review did not find a significant increase in gestational diabetes, pregnancy induced hypertension and postpartum haemorrhage in the pandemic compared with the pre-pandemic period, but found a significant increase in surgical management of ruptured ectopic pregnancy [3]. The studies from India did not report changes in the incidence and deaths due to severe maternal complications.[4,5] Therefore, the objectives of this study were to compare the incidence and case-fatality of severe life-threatening maternal complications in India on a larger population base between the SARS-CoV-2 pandemic and the pre-pandemic periods, and to examine trends across the two pandemic waves.

## 2 Methods

Study design: A hospital-based repeated monthly survey of severe maternal complications and death is being undertaken through the Maternal and perinatal Health Research collaboration, India (MaatHRI) [11]. The survey has been ongoing since August 2018 in 15 selected public and private hospitals in five Indian states: Assam, Maharashtra, Uttar Pradesh, Himachal Pradesh and Meghalaya. The data included in this study covers a total of 202,986 hospital births over a period of 30 months, December 2018 through to May 2021, 15 months pre-pandemic and 15 months during the pandemic. All co-authors in the writing group had access to the data.

Data collection: A method tested in a pilot study in India [12] has been employed to conduct an ongoing monthly survey of nine severe maternal complications: (i) postpartum haemorrhage (PPH), (ii) eclampsia, (iii) pre-eclampsia, (iv) septic abortion (sepsis following spontaneous or induced abortion), (v) maternal peripartum infection (not abortion related), (vi) uterine rupture, (vii) heart failure in pregnancy or postpartum, (viii) transient peripheral neuropathy during pregnancy or postpartum and (ix) Japanese encephalitis. In addition, hospitals are encouraged to report other severe complications not included on the list. Standard definitions are used for each condition (supplementary file, Table-S1). If a standard definition was not available for a particular condition, for example heart failure and transient peripheral neuropathy in pregnant or postpartum women, a working definition was developed by expert clinicians in conjunction with the study collaborators and MaatHRI steering committee [11]. In cases of multiple co-morbidities, the primary complication is ascertained, which is defined as the complication that led to other co-morbidities. For example, if a woman had sepsis that led to disseminated intravascular coagulation (DIC) and ultimately death from haemorrhage, the primary maternal complication was recorded as Sepsis.

Data on the number of clinically confirmed cases and deaths due to each condition and total number of births are collected monthly from the hospital records by a research nurse and reported through an electronic case reporting system. Completeness is ensued through ‘nil’ reporting. A copy of the case notification form is included in the supplementary file (Appendix-1). During a few months at the start of the pandemic in March 2020, there was a slight delay in reporting, but the reports were always complete and data quality checks were done every month to identify any inaccuracies.

The reporting period from December 2018 to May 2021 was divided into pre-pandemic (Dec-18 to Feb-2020) and pandemic (Mar-20 to May-21) periods based on the data from the Johns Hopkin's Resource Centre pertaining to the Coronavirus pandemic waves in India [1]. Using this information, the pandemic period was further divided into three phases: ‘pandemic-wave1 rising (Mar-Sep 2020)’, ‘pandemic-wave1 receding (Oct20-Feb21)’ and ‘pandemic-wave2 rising (Mar-May 2021)’. We used the time series data on the Government Response Stringency Index (GRSI) for India, January 2020 through to May 2021, to calculate monthly average GRSI scores and average scores corresponding with the phases of the pandemic wave. The GRSI is a daily score computed since January 2020 by the Blavatnik School of Government at the University of Oxford (Oxford, UK) based on the strictness of lockdown and restrictions implemented by a given country to mitigate SARS-CoV-2 outbreaks [13].

Statistical analysis: Incidence rates were defined as the number of new severe maternal complications in the study hospitals per 1000 hospital births over a given period of time, and Poisson incidence rates (with 95% Confidence Intervals (CIs)) [14] were calculated. Case-fatality was defined as the proportion of women with newly diagnosed severe maternal complications who died within a given time-period (i.e. the number died as a proportion of the number newly diagnosed), and reported as percentage of cases with 95% CIs. Incidence and case-fatality were calculated for overall complications and individually for the following specific complications: (i) PPH, (ii) eclampsia, (iii) pre-eclampsia, (iv) maternal peripartum infection, (v) septic abortion, (vi) uterine rupture, (vii) heart failure in pregnancy or postpartum, and (viii) others which included transient peripheral neuropathy during pregnancy or postpartum, Japanese encephalitis, and other severe complications such as antepartum haemorrhage, pulmonary oedema, jaundice in pregnancy, gestational diabetes mellitus, and ruptured ectopic pregnancy. Due to small numbers, transient peripheral neuropathy and Japanese encephalitis were grouped along with the other complications.

We calculated the percentage change in hospital births per month during the pandemic period compared with the same calendar month in the preceding pre-pandemic period. Linear regression accounting for time-period was used to examine the association between GRSI scores and change in hospital births, overall incidence and case-fatality per month. Fractional polynomials were used to examine the presence of non-linear associations between GRSI scores and the outcome variables. Appropriateness of the linear models were further examined by plotting the residuals. The β-coefficients and GRSI scores were scaled to interpret the results as ‘per 10%’ increase in the GRSI scores.

We compared the incidence and case-fatality in the pandemic period with the pre-pandemic period by calculating rate ratios (RR) with 95% CIs and also graphically plotted the rates against the average GRSI score for the corresponding phase. RR with 95% CIs for overall and specific incidence and case-fatality were calculated to compare each phase of the pandemic wave with the pre-pandemic period. Where there were small number of events we did not calculate incidence and case-fatality rates within States, but we conducted sub-group analysis to examine the trends across the five States. All analyses were adjusted for clustering at the level of the hospitals using Robust Standard Errors. Results were considered to be significant at a p-value of <0.05. Stata version 16.1 SE (StataCorp, College Station, Texas) was used to conduct all analyses and graphs were plotted using Microsoft Excel.

### 2.1 Ethics approval

The MaatHRI platform and the repeated monthly survey have been approved by the institutional review boards (IRB) of each coordinating Indian institution, namely: Srimanta Sankaradeva University of Health Sciences, Guwahati, Assam (No.MC/190/2007/Pt-1/126); Nazareth hospital, Shillong, Meghalaya (Ref No. NH/CMO/IEC/COMMUNICATIONS/18-01); Emmanuel Hospital Association, New Delhi (Ref. Protocol No.167); Mahatma Gandhi Institute of Medical Sciences, Sevagram, Maharashtra (Ref No. MGIMS/IEC/OBGY/118/2017); and the Institute of Medical Sciences, Banaras Hindu University, Varanasi, Uttar Pradesh (No.Dean/2018/EC/290). The project has also been approved by the Government of India's Health Ministry's Screening Committee, the Indian Council of Medical Research, New Delhi (ID number 2018-0152) and by the Oxford Tropical Research Ethics Committee (OxTREC), University of Oxford, UK (OxTREC Ref: 7–18).

Patient/ participant consent: Not applicable as these are aggregate numbers of cases and deaths from hospital records.

Role of the funding source: The MaatHRI platform and this study are funded by a Medical Research Council Career Development Award to MN (Ref:MR/P022030/1). The funder has no role in the study design, data collection, analysis, or writing the paper.

## 3 Results

There were 202,986 hospital births during the 30 months of the study period; 113,140 in the 15 months of the pre-pandemic period and 89,846 in the 15 months of the pandemic period. A total of 24,109 women were admitted with at least one of the nine surveyed severe maternal complications and a further 869 with other severe complications over the 30 months. A total of 1020 women in the study population died. Thus, the overall incidence rate for severe maternal complications in the 15 hospitals across the five Indian States was 123.05 per 1000 hospital births (95% CI 90.40 to 167.51) and about 1 in 25 women who presented with a complication died (case-fatality 4.08%, 95% CI 2.87 to 5.81).

**Fig. 1:**
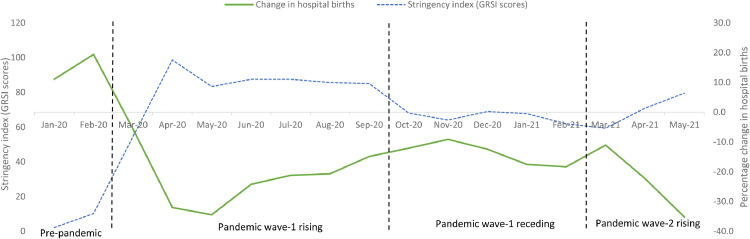
Change in hospital births in the pandemic period compared with the same month in the preceding pre-pandemic period compared with the stringency index (GRSI scores, January 2020* through May 2021) in India. Data source for the change in births: MaatHRI; Data source for the Stringency index: Government Response Stringency Index (GRSI) developed by the Blavatnik School of Government at the University of Oxford (Oxford, UK); *GRSI scores available from January 2020 onwards.

There was a significant decrease in hospital births per month during the pandemic compared with the same month in the preceding pre-pandemic period (Fig. 1). The study showed that births in the 15 study hospitals across five states in India decreased by 4.8% per 10% increase in the Indian Government Response Stringency Index (GRSI). (*p* < 0.001). Hospital births decreased by more than 30% during the start of the first wave of the pandemic and increased gradually towards the end of the first wave, but decreased again during the ongoing second wave with more than a 35% decrease in May 2021 compared with May 2020. Hospital births never reached the pre-pandemic levels since the start of the pandemic and social restrictions in March 2020. There was also a statistically significant positive linear association between the GRSI scores and overall incidence rate driven mainly by the decrease in hospital births (change in incidence of 3.3 per 1000 births per 10% increase in GRSI scores, 95% CI 1.5 to 5.5 per 1000 births, *p* = 0.002). The case-fatality was not significantly associated with the GRSI scores (change in case-fatality of 0.2% per 10% increase in GRSI scores, 95% CI −0.1% to 0.4%, *p* = 0.120) (Figs. S1 and S2, supplementary file). We did not find significant non-linear associations between the GRSI scores and the outcome variables and the plotted residuals from the regression analyses showed a normal distribution suggesting a linear association.

Comparing the pandemic period with the pre-pandemic period (Table-1) showed that the overall incidence of maternal complications increased from 117.93 per 1000 to 129.50 per 1000 hospital births (RR = 1.10, 95% CI = 0.89–1.36, *p* = 0.386), and the trend was similar across the five study States. The overall case-fatality increased by 23% (RR = 1.23, 95% CI = 1.03–1.46, *p* = 0.022) from 3.69% in the pre-pandemic period to 4.53% in the pandemic period, but the case-fatality was mainly significantly higher in Assam (RR = 1.26; 95% CI = 1.06–1.50; *p* = 0.011). The RR for case-fatality in Uttar Pradesh was 1.78; 95% CI = 0.96–3.28; *p* = 0.065 and in the other three States was 0.38; 95%CI = 0.12–1.21; *p* = 0.102. Although the overall incidence rate in the pandemic period was not statistically significantly higher than the incidence in the pre-pandemic period, the incidence rate of septic abortion was 56% higher (RR = 1.56, 95% CI = 1.22–1.99, *p* < 0.001). There was a 24% increase in the total number of hospital admissions for septic abortion cases during the pandemic period compared with the pre-pandemic period. There was a statistically significant linear association between incidence of septic abortion and GRSI scores with a change in incidence of 1.2 per 1000 births per 10% increase in GRSI scores, 95% CI 0.2 to 2.3 per 1000 births, *p* = 0.022. We also found a two-fold increase in the case-fatality for heart failure increasing from 28.9 to 58.2% (RR = 2.01, 95% CI = 1.53–2.65, *p* < 0.001).

**Table 1:** Severe maternal complications, incidence and death in 15 study hospitals during the pandemic and pre-pandemic periods in India, December 2018 through May 2021

	Incidence of severe maternal complications				Case-fatality			
Severe maternal complications	Incidence rate per 1000 hospital births (N) Pre-pandemic	Incidence rate per 1000 hospital births (N) Pandemic period	Rate ratio (95% CI) (pandemic period compared with the pre-pandemic)	*p*-value for the rate ratio	Case-fatality Pre-pandemic% (N)	Case-fatality Pandemic period% (N)	Rate ratio (95% CI) (pandemic period compared with the pre-pandemic period)	*p*-value for the rate ratio
All complications	117.93 (13,343)	129.50 (11,635)	1.10 (0.89 – 1.36)	0.386	3.69 (493)	4.53 (527)	1.23 (1.03 – 1.46)	0.022
Specific complications								
Eclampsia	15.27 (1728)	15.65 (1406)	1.02 (0.80 – 1.31)	0.848	6.08 (105)	7.82 (111)	1.29 (0.77 – 2.16)	0.339
Pre-eclampsia	68.61 (7762)	73.44 (6598)	1.07 (0.78 – 1.47)	0.674	5.93 (46)	5.76 (38)	0.97 (0.40 to 2.56)	0.954
Postpartum haemorrhage	12.82 (1451)	13.50 (1213)	1.05 (0.76 – 1.46)	0.759	4.96 (72)	5.85 (71)	1.18 (0.78 – 1.78)	0.429
Septic abortion	5.81 (657)	9.05 (813)	1.56 (1.22 – 1.99)	<0.001	3.65 (24)	3.44 (29)	0.94 (0.30 – 2.92)	0.919
Maternal peripartum infection (not abortion related)	5.34 (604)	7.27 (653)	1.36 (0.77 – 2.41)	0.289	12.58 (76)	11.33 (74)	0.90 (0.38 – 2.14)	0.813
Heart failure*	2.26 (256)	2.10 (189)	0.93 (0.59 – 1.46)	0.750	28.91 (74)	58.20 (110)	2.01 (1.53 – 2.65)	<0.001
Uterine rupture	2.88 (326)	2.34 (210)	0.81 (0.56 – 1.17)	0.264	4.60 (15)	6.67 (14)	1.45 (0.87 – 2.42)	0.156
Other complications	4.94 (559)	6.16 (553)	1.25 (0.71 – 2.18)	0.442	14.31 (81)	14.47 (80)	1.01 (0.46 – 2.24)	0.979

Total births in the study hospitals in the pre-pandemic period = 113,140; Total births in the study hospitals in the pandemic period = 89,846; p-value adjusted for clustering at the level of the hospitals; *Heart failure during pregnancy and postpartum; Other complications include transient peripheral neuropathy during pregnancy or postpartum, Japanese encephalitis, antepartum haemorrhage, pulmonary oedema, jaundice in pregnancy, gestational diabetes mellitus, and ruptured ectopic pregnancy.

**Fig. 2:**
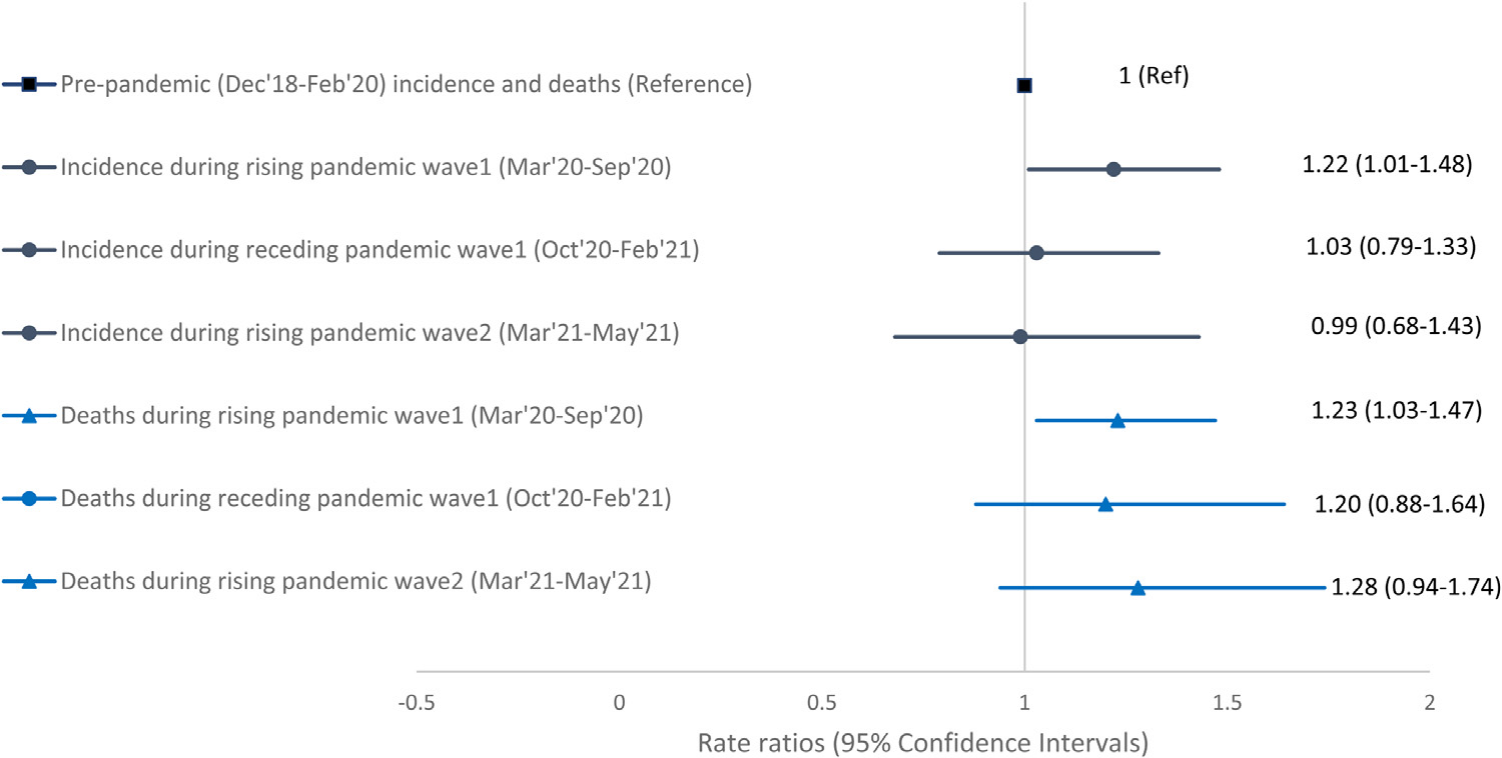
Hospital-based incidence and case-fatality of severe maternal complications during the pandemic waves compared with the pre-pandemic period in India (December 2018 through May 2021).

**Fig. 3:**
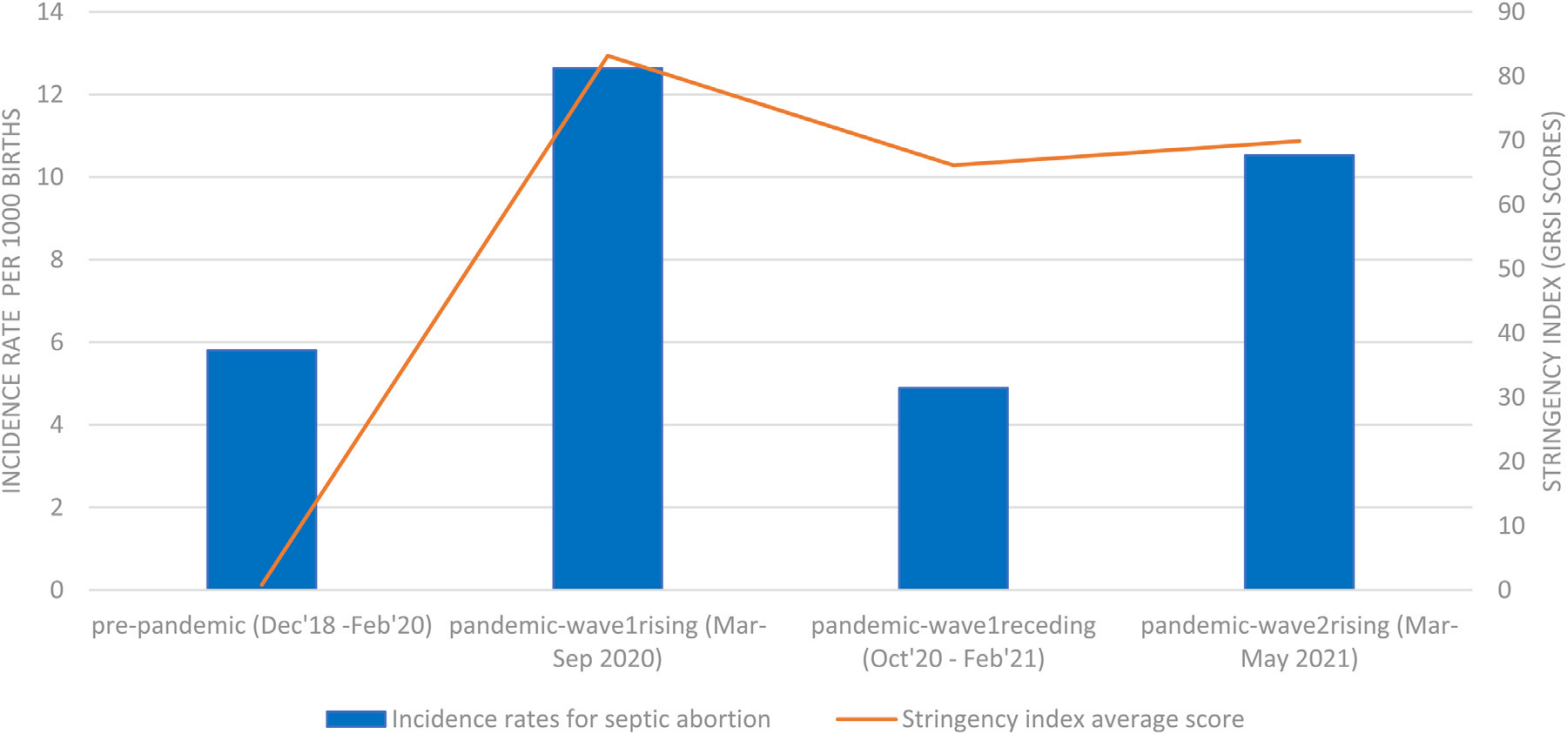
**Incidence of septic abortion across the phases of the pandemic in India, December 2018 through May 2021.** Test for heterogeneity in incidence rates of septic abortion across the phases of the pandemic, p-value <0.001; Change in incidence of septic abortion by 1.2 per 1000 births per 10% increase in GRSI scores after accounting for time, 95% CI 0.2 to 2.3 per 1000 births, *p* = 0.022; Data source for the incidence of septic abortion: MaatHRI; Data source for the Stringency index: Government Response Stringency Index (GRSI) developed by the Blavatnik School of Government at the University of Oxford.

A detailed examination of the phases of the pandemic period (wave-1 rising, wave-1 receding and wave-2 rising) showed that compared with the pre-pandemic period, the overall incidence of severe maternal complications increased significantly by 22% when the first wave was rising; *p*-value = 0.044 (Fig.-2) and followed closely with the average GRSI scores for the different phases of the pandemic. The incidence rates were not significantly different from the pre-pandemic rates for the other two phases of the pandemic; *p*-values 0.843 and 0.944 for wave-1 receding and wave-2 rising, respectively (Fig.-2). With regard to specific complications, the incidence rates did not differ across the phases of the pandemic compared with the pre-pandemic period except for septic abortion rates which were two-fold higher than the pre-pandemic rates during wave-1 rising (RR = 2.18; 95% CI = 1.66–2.85, *p* < 0.001) with a similar trend during wave-2 rising with only 3 months of data available (Fig.-3).

**Fig. 4:**
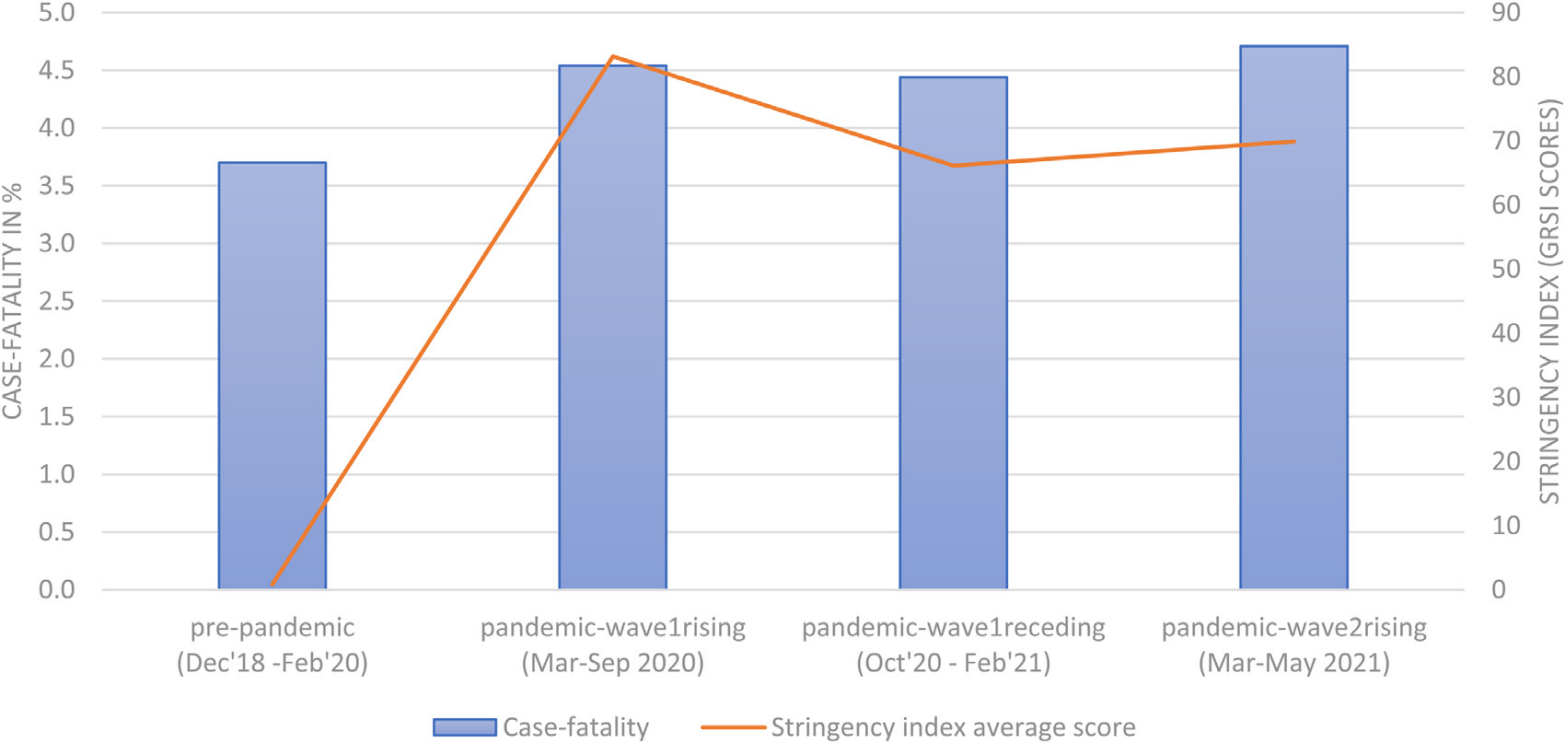
**Case-fatality of severe maternal complications across the phases of the pandemic in India, December 2018 through May 2021.** Test for heterogeneity in case-fatality across the phases of the pandemic, *p*-value = 0.103;Change in case-fatality of 0.2% per 10% increase in GRSI scores after accounting for time, 95% CI −0.1% to 0.4%, *p* = 0.120;Data source for case-fatality: MaatHRI; Data source for the Stringency index: Government Response Stringency Index (GRSI) developed by the Blavatnik School of Government at the University of Oxford.

The case-fatality figures were 20–28% higher across the phases of the pandemic compared with the pre-pandemic period (Fig.-2) and remained high despite a decrease in the GRSI scores (Fig.-4). These were not statistically significantly higher during wave-1 receding and wave-2 rising possibly due to the smaller sample. Case-fatality varied across the categories for heart failure during pregnancy or postpartum (Table-2), being significantly higher across all phases of the pandemic compared with the pre-pandemic period; *p* < 0.001 for wave-1 rising and receding and *p* = 0.001 for wave-2 rising. Case-fatality for uterine rupture was nearly 2.5 times higher during wave-2 rising compared with the pre-pandemic period (RR = 2.47, 95% CI = 0.99–6.17, *p* = 0.053).

**Table 2:** Rate ratios of incidence and case-fatality of severe maternal complications in 15 study hospitals across the different phases of the pandemic in India, December 2018 through May 2021

Phases of the pandemic period	Eclampsia RR (95% CI)	Pre-eclampsia RR (95% CI)	Postpartum haemorrhage RR (95% CI)	Septic abortion RR (95% CI)	Maternal peripartum infection (not abortion related) RR (95% CI)	Heart failure* RR (95% CI)	Uterine rupture RR (95% CI)	Other complications RR (95% CI)
**Incidence**								
Pre-pandemic (Dec'18 -Feb'20)	1 (ref)	1 (ref)	1 (ref)	1 (ref)	1 (ref)	1 (ref)	1 (ref)	1 (ref)
Pandemic-wave-1 rising (Mar-Sep 2020)	1.14 (0.77 – 1.69)	1.16 (0.85 – 1.59)	1.26 (0.81 – 1.97)	2.18 (1.66 – 2.85)	1.37 (0.88 – 2.14)	1.02 (0.59 – 1.75)	0.86 (0.56 – 1.33)	1.20 (0.74 – 1.96)
Pandemic-wave-1 receding (Oct'20 - Feb'21)	1.03 (0.83 – 1.27)	1.07 (0.74 – 1.54)	0.88 (0.63 – 1.22)	0.84 (0.49- 1.45)	1.15 (0.71 – 1.85)	0.91 (0.57 – 1.47)	0.71 (0.50 – 1.00)	1.19 (0.61 – 2.32)
Pandemic-wave-2 rising (Mar-May 2021)	0.76 (0.56 – 1.03)	0.88 (0.61 – 1.27)	0.98 (0.68 – 1.43)	1.81 (0.84 – 3.93)	1.82 (0.44 – 7.55)	0.76 (0.49 – 1.18)	0.94 (0.56 – 1.57)	1.47 (0.66 – 3.29)
**Case-fatality**								
Pre-pandemic (Dec'18 -Feb'20)	1 (ref)	1 (ref)	1 (ref)	1 (ref)	1 (ref)	1 (ref)	1 (ref)	1 (ref)
Pandemic-wave-1 rising (Mar-Sep 2020)	1.25 (0.64 – 2.43)	1.11 (0.49 – 2.51)	1.13 (0.71 – 1.81)	0.83 (0.24 – 2.89)	0.80 (0.38 – 1.71)	2.38 (1.60 – 3.56)	1.45 (0.81 – 2.58)	1.00 (0.59 – 1.71)
Pandemic-wave-1 receding (Oct'20 - Feb'21)	1.35 (0.70- 2.63)	0.87 (0.21 – 3.55)	1.20 (0.52 – 2.80)	1.20 (0.28 – 5.25)	1.15 (0.44 – 2.97)	1.71 (1.37 – 2.13)	0.86 (0.31 – 2.40)	0.99 (0.46 – 2.12)
Pandemic-wave-2 rising (Mar-May 2021)	1.23 (0.71 – 2.12)	0.86 (0.43 – 1.73)	1.28 (0.83 – 1.98)	0.96 (0.31 – 2.95)	0.70 (0.17 – 2.97)	1.73 (1.27 – 2.36)	2.47 (0.99 – 6.17)	1.07 (0.22 – 5.25)

Total births in the study hospitals in the pre-pandemic period = 113,140; Total births in the study hospitals in the pandemic period wave-1 rising = 36,382; Total births in the study hospitals in the pandemic period wave-1 receding = 37,231; Total births in the study hospitals in the pandemic period wave-2 rising = 16,233; RR-rate ratio.

⁎ Heart failure during pregnancy and postpartum; Other complications include transient peripheral neuropathy during pregnancy or postpartum, Japanese encephalitis, antepartum haemorrhage, pulmonary oedema, jaundice in pregnancy, gestational diabetes mellitus, and ruptured ectopic pregnancy.

## 4 Discussion

The study showed a fall in hospital births by about 5% per 10% increase in stringency index (GRSI scores). The overall hospital-level incidence rate for severe maternal complications increased by 10% in the pandemic period compared with the pre-pandemic period, but this was mainly driven by a significant increase in rate of hospital admissions from septic abortion, which was two-fold higher during the first and second waves of the pandemic. The overall hospital case-fatality increased by 23% compared with the pre-pandemic period and remained high across the different phases of the pandemic. There was a notably high rate of death amongst women with uterine rupture and heart failure during pregnancy or postpartum.

The decrease in hospital births corresponding with an increase in stringency index suggests that lockdown and other restrictions had a strong negative impact on institutional birth in the study settings in India. This conforms to the findings of two previous studies by Kumari et al [5]. and Goyal et al. [4], each in a single tertiary hospital in India, which respectively reported a 43% and 45% reduction in hospital births during the first wave of the pandemic compared with the pre-pandemic period. This has serious implications for access to care at childbirth and threatens to reverse the gains made in increasing institutional births and access to emergency obstetric services in India, [15] which are important determinants to reduce maternal mortality.

There was no observed significant change in the incidence of severe maternal complications, except septic abortion, during the pandemic period. We observed a decrease in the overall number of cases (except septic abortion) and hospital births; it seems likely that mostly very sick women were coming to hospital. The relative increase in case-fatality was due to an increase in deaths from all surveyed complications, heart failure being significantly higher. Case-fatality increased during the start of the first wave and remained high throughout the different phases of the pandemic irrespective of the stringency index/GRSI scores in India. Kumari et al [5]. found a 7% increase in maternal deaths in a tertiary hospital during the strict lockdown period in wave-1, but Goyal et al [4]. did not find any significant increase during the first phase of the pandemic. The systematic review did not find any association between maternal, perinatal and neonatal outcomes and the GRSI scores, and concluded that stringent government response had no effect, rather the increase in adverse outcomes were attributed to inefficiency of healthcare systems [3]. In contrast to other studies that covered a short duration of the pandemic, our study presents the effects across different phases of the pandemic and restrictions over a longer duration in a much larger sample of 202,986 hospital births. The evidence generated here suggests that the negative impacts on maternal and reproductive health was much higher during the rising first wave corresponding with the period of strictest lockdown and major disruptions in India and a similar trend can be seen during the ongoing second wave.

The high case-fatality could be due to a number of factors, including reductions in healthcare providers and medical supplies as a result of reallocation to mitigate the SARS-CoV-2 pandemic or a decrease in access and/or demand for healthcare services [2]. While the quality of care in the hospitals during the pandemic could have been compromised due to shortage of healthcare staff, converting hospital wards into SARS-CoV-2 treatment Units, and other administrative challenges [16] we cannot ignore access and demand as being a major risk factor for the increase in maternal deaths in the hospitals both due to a lack of transportation during lockdowns and ‘hospital-avoiding’ behaviour by pregnant women suggested by other studies [4,5,17].

The negative impact of the pandemic and related restrictions on sexual and reproductive health has been further demonstrated by an increase in hospital admission rates of septic abortion confirming the fears about increase in unsafe abortion raised by international organisations like the International Federation of Gynaecology and Obstetrics (FIGO) [8] and Marie Stopes International[7] at the start of the pandemic. Unsafe abortion is the third leading cause of maternal mortality in India [7]. In a previous study covering nine states in India, we found that two-thirds of the abortions were unsafe [18]. An overall 56% increase in the rate of septic abortion with a two-fold increase corresponding with the periods of rising first and second waves suggest that access to safe abortion services were further compromised during the pandemic, particularly during the periods of the strictest lockdown in India. Evidence related to incidence of severe maternal complications from the other two studies were mixed, with Goyal et al [4]. reporting 7% increase in hospital admissions of high-risk pregnancies and Kumari et al [5]. reporting 66% decrease in obstetric emergencies. We did not find any study from India that specifically examined the incidence and deaths from unsafe abortion during the pandemic. The estimated 23% increase in deaths from severe maternal complications and two-fold increase in hospital admissions from septic abortion observed in the study hospitals are only the tip of the ice-berg. The overall maternal mortality and morbidity rates in hospitals and communities could be much higher.

This is the first large study from a hospital-based survey across five states in India comparing the incidence and case-fatality of severe maternal complications in more than 202,000 births during the pandemic and pre-pandemic periods over a duration of 30 months. We were also able to compare the trends across the different phases of two waves of the pandemic. We did not have information on maternal hospital admissions and death rates due to complications other than those included in the study, therefore the reported case-fatality do not reflect the overall maternal mortality rate in the population. We also did not have additional information about SARS-CoV-2 infection in the cases and deaths included in the study, and therefore cannot ascertain any additional effect of this co-morbidity on case-fatality. This was beyond the scope of this paper and further epidemiological studies are required to delineate the effect.

We acknowledge that given the rarity of most of the outcomes explored, there are limits to the statistical power to detect small changes in these outcomes. Although we had a large sample of hospital births, the number of cases and deaths for each condition was still small which together with accounting for clustering limited the power to detect statistically significant RR. It is important to note that our study hospitals are from five States in India with a varying pre-existing burden of maternal mortality. The relative increase in case-fatality was higher in the two states that have a high burden (Assam and Uttar Pradesh) than the three that have a lower burden (Meghalaya, Maharashtra and Himachal Pradesh) [19], thus the overall rate would be generalizable to the country. Furthermore, the collaborating hospitals are both from the public and the private sectors. A majority are tertiary level referral facilities except two which are community level hospitals. These are representative of the different types of referral hospitals that cater to the population across India. Further details about the study hospitals can be found in the MaatHRI methodology paper [11] and current updates are available on the MaatHRI website https://www.npeu.ox.ac.uk/maathri.

Our study supports the legitimacy of the calls made by the WHO, other international organisations and scientists to maintain sexual and reproductive health services as essential services to continue to provide high quality care in order to avert rise in maternal mortality and morbidity during the pandemic [7,8]. The evidence supports the recommendations made by other studies in India to encourage women to seek timely care by alleviating fears of catching SARS-CoV-2 infection in the hospital [4,5,16]. While India is preparing to manage a third wave of the pandemic, the country needs to take urgent action to mitigate the ongoing reproductive health crisis using the lessons learnt from the first and second waves. Special transport/ambulance services could be made available for pregnant women during periods of lockdowns together with active public health messaging urging women to seek required reproductive health care including safe abortion services. Since 2000, there has been an estimated 5.5% average annual decline in the maternal mortality ratio in India [20], but if the observed trend of 20–28% increase in case-fatality continues, it could push back the progress made by several years. Furthermore, there are wider implications of the pandemic on reproductive health affecting access to safe abortion services, and if allowed to continue, could lead to devastating consequences for thousands of women, their families, and the society. It will hinder and most likely reverse the progress made in achieving the Sustainable Development Goal of reducing maternal mortality in India and globally.

The MaatHRI collaboration will continue to monitor the effects of the pandemic on the incidence and case-fatality of severe maternal complications in the 15 hospitals across the country and updates on the trend will be henceforth disseminated regularly through the MaatHRI quarterly newsletter available through https://www.npeu.ox.ac.uk/maathri/newsletters.

## Author contributions

The corresponding and senior author MN developed the concept for the study, led the work as the chief investigator, analysed the data, and wrote the first draft of the paper. OB, AKB, SC, SCC, AC, BD, GD, PJ, SDK, PK, PM, RM, AR, SR, IR, RKT, CSV, ST, AV, and FZ contributed equally, and their names are included in the alphabetic order of their last name. They are obstetricians and are collaborators and investigators of the MaatHRI project who led the work in their respective institutions. They contributed to the concept, supervised data collection, interpreted the findings, and edited the paper. RD is the MaatHRI Project Manager who supervised data collection and edited the paper. CO is a bio-statistician and provided statistical support for the analysis and edited the paper. JJK contributed to the concept and edited the paper.

## Funding

The MaatHRI platform and this study are funded by a Medical Research Council Career Development Award to MN (Ref:MR/P022030/1). The funder has no role in the study design, data collection, analysis, or writing the paper.

## Data sharing statement

Data included in the paper is available for free and can be obtained by contacting the corresponding author.

## Declaration of Competing Interest

Manisha Nair declares a Medical Research Council Career Development Award (Ref:MR/P022030/1), including support for attending meetings and/or travel.

## Appendix Supplementary materials

Image, application 1

Image, application 2
