# Supplementary material for: Reproductive health crisis during waves one and two of the COVID-19 pandemic in India: Incidence and deaths from severe maternal complications in more than 202,000 hospital births
Source: eClinicalMedicine. 2021 Jul 29;39:101063. doi: 10.1016/j.eclinm.2021.101063 (PMC8461242; doi:10.1016/j.eclinm.2021.101063)
Supplement: Supplementary file 1 [file mmc1.docx]

**MaatHRI writing group** on behalf of the MaatHRI collaborators, <https://www.npeu.ox.ac.uk/maathri>

Manisha Nair (MN), National Perinatal Epidemiology Unit, Nuffield Department of Population Health, University of Oxford, Oxford, UK;

Omesh Bharti (OB), State Institute of Health and Family Welfare,  Department of Health & Family Welfare, Government of Himachal Pradesh, India;

Amrit Krishna Bora (AKB), Mahendra Mohan Choudhury Hospital, Assam, India;

Shakuntala Chhabra (SC), Mahatma Gandhi Institute of Medical Sciences, Maharashtra, India;

Saswati S Choudhury (SSC), Gauhati Medical College and Hospital, Assam, India;

Bandana Das (BD), Silchar Medical College and Hospital, Assam, India;

Gitanjali Deka (GD), Tezpur Medical College, Assam, India;

Punam Jain (PJ), Nazareth Hospital, Meghalaya, India;

Swapna D Kakoty (SDK), Fakhruddin Ali Ahmed Medical College and Hospital, Assam, India;

Pramod Kumar (PK), Mahatma Gandhi Institute of Medical Sciences, Maharashtra, India;

Pranabika Mahanta (PM), Jorhat Medical College and Hospital, Assam, India;

Robin Medhi (RM), Fakhruddin Ali Ahmed Medical College and Hospital, Assam, India;

Anjali Rani (AR), Institute of Medical Sciences, Banaras Hindu University, Uttar Pradesh, India;

Sereesha Rao (SR), Silchar Medical College and Hospital, Assam, India;

Indrani Roy (IR), Nazareth Hospital, Meghalaya, India;

Ratna Kanta Talukdar (RKT), Jorhat Medical College and Hospital, Assam, India;

Carolin Solomi V (CSV), Makunda Christian Leprosy and General Hospital, Assam, India;

Sita Thakur (ST), Dr Rajendra Prasad Government Medical College Kangra at Tanda, Himachal Pradesh, India

Ashok Verma (AV), Dr Rajendra Prasad Government Medical College Kangra at Tanda, Himachal Pradesh, India

Farzana Zahir (FZ), Assam Medical College, Assam, India;

Rupanjali Deka (RD), Project Manager, MaatHRI, Assam, India

Charles Opondo (CO), National Perinatal Epidemiology Unit, Nuffield Department of Population Health, University of Oxford, Oxford, UK

Jennifer J Kurinczuk (JJK), National Perinatal Epidemiology Unit, Nuffield Department of Population Health, University of Oxford, Oxford, UK
