# Supplementary material for: Reproductive health crisis during waves one and two of the COVID-19 pandemic in India: Incidence and deaths from severe maternal complications in more than 202,000 hospital births
Source: eClinicalMedicine. 2021 Jul 29;39:101063. doi: 10.1016/j.eclinm.2021.101063 (PMC8461242; doi:10.1016/j.eclinm.2021.101063)
Supplement: Supplementary file 2 [file mmc2.docx]

**Table-S1: Standard definition of health conditions included in the survey**

| Condition | Definition |
| --- | --- |
| Postpartum haemorrhage (WHO)^1^ | A pregnant woman having a blood loss of ≥500 ml from the genital tract during or within 24 hours of childbirth. |
| Pre-eclampsia (NICE & WHO)^23^ | A pregnant woman with:  onset of a new episode of hypertension during pregnancy  AND/ OR  occurrence of substantial proteinuria (>0.3 g/24 h) |
| Eclampsia (WHO)^2^ | A pregnant woman with:  onset of a new episode of hypertension during pregnancy (with persistent diastolic blood pressure >90 mm Hg)  AND  occurrence of generalized seizures, provided that the tonic–clonic seizures are not attributable to other causes (e.g. epilepsy)  AND/ OR  occurrence of substantial proteinuria (>0.3 g/24 h) |
| Maternal peripartum infection (WHO definition)^4^ | Maternal peripartum infection is defined as bacterial infection of the genital tract or its surrounding tissues occurring at any time between the onset of rupture of membranes or labour and the 42nd day postpartum in which two or more of the following are present:   - Pelvic pain - Fever - Abnormal vaginal discharge - Abnormal smell/foul odour discharge - Delay in uterine involution |
| Septic abortion (Cochrane Database of Systematic Reviews 2016)^5^ | Any abortion with infection after a miscarriage or intentional pregnancy termination. |
| Uterine rupture (UKOSS)^6^ | A complete separation of the wall of the pregnant uterus, with or without expulsion of the fetus, involving rupture of membranes at the site of the uterine rupture or extension into uterine muscle separate from any previous scar. |
| Heart failure in pregnant or postpartum women due to any causes (Working definition adapted from a review on ‘Decompensated Heart Failure in Pregnancy’)^7^ in consultation with cardiologists | Pregnant or postpartum women –   - Suspected of having a heart failure by the attending clinician   OR   - Presenting with breathlessness (≥15 breaths per minute)   With or without one or more of the following signs   - Elevated Jugular venous pressure - A cardiac murmur – gallop rhythm - Signs of pulmonary oedema (crackles in the lung) |
| Transient peripheral neuropathy (WHO definition for Thiamine deficiency with peripheral neuropathy)^8^ | A pregnant woman presenting with polyneuropathy with paraesthesia of the extremities (especially the legs), reduced knee jerk and other tendon reflexes, and progressive severe weakness and wasting of muscles. |
| Japanese Encephalitis complication in pregnancy | A pregnant women suspected to have a Japanese Encephalitis infection. |

Reference list for Table-S1

1. WHO. WHO recommendations for the prevention and treatment of postpartum haemorrhage. Italy: WHO 2012.

2. World Health Organisation. WHO recommendations for prevention and treatment of pre-eclampsia and eclampsia. Geneva: WHO Press, World Health Organization 2011.

3. Brown MA, Magee LA, Kenny LC, Karumanchi SA, McCarthy FP, Saito S, et al. The hypertensive disorders of pregnancy: ISSHP classification, diagnosis & management recommendations for international practice. Pregnancy Hypertension. 2018;13:291-310.

4. World Health Organisation. WHO recommendations for prevention and treatment of maternal peripartum infections. Geneva: WHO Press, World Health Organization 2015.

5. Udoh A, Effa EE, Oduwole O, et al. Antibiotics for treating septic abortion. *Cochrane Database of Systematic Reviews* 2016(7) doi: 10.1002/14651858.CD011528.pub2

6. Fitzpatrick KE, Kurinczuk JJ, Alfirevic Z, et al. Uterine rupture by intended mode of delivery in the UK: a national case-control study. *PLoS medicine* 2012;9(3):e1001184.

7. Anthony J, Sliwa K. Decompensated Heart Failure in Pregnancy. *Cardiac Failure Review* 2016;2(1):20-26. doi: 10.15420/cfr.2015:24:2

8. World Health Organisation. Thiamine deficiency and its prevention and control in major emergencies. Geneva: WHO Press, World Health Organisation 1999.

**Appendix-1: MaatHRI monthly case notification form**

Figure S1: Monthly comparison of incidence rates of severe maternal complications and stringency index (GRSI scores, January 2020* through May 2021) in India

Change in incidence of 3.3 per 1000 births per 10% increase in GRSI scores after accounting for time, 95% CI 1.5 to 5.5 per 1000 births, p=0.002Data source for the change in births: MaatHRI;

Data source for the Stringency index: Government Response Stringency Index (GRSI) developed by the Blavatnik School of Government at the University of Oxford (Oxford, UK); *GRSI scores available from January 2020 onwards

Figure S2: Monthly comparison of case-fatality of severe maternal complications and stringency index (GRSI scores, January 2020* through May 2021) in India

Change in case-fatality of 0.2% per 10% increase in GRSI scores after accounting for time, 95% CI -0.1 to 0.4%, p=0.120

Data source for the change in births: MaatHRI;

Data source for the Stringency index: Government Response Stringency Index (GRSI) developed by the Blavatnik School of Government at the University of Oxford (Oxford, UK); *GRSI scores available from January 2020 onwards
